# On-site and visual detection of sorghum mosaic virus and rice stripe mosaic virus based on reverse transcription-recombinase-aided amplification and CRISPR/Cas12a

**DOI:** 10.3389/fgeed.2023.1124794

**Published:** 2023-01-20

**Authors:** Junkai Wang, Xiuqin Huang, Siping Chen, Jiahao Chen, Zhengyi Liang, Biao Chen, Xin Yang, Guohui Zhou, Tong Zhang

**Affiliations:** ^1^ Guangdong Province Key Laboratory of Microbial Signals and Disease Control, College of Plant Protection, South China Agricultural University, Guangzhou, China; ^2^ State Key Laboratory for Conservation and Utilization of Subtropical Agro-Bioresources, South China Agricultural University, Guangzhou, China

**Keywords:** RT-RAA, CRISPR/Cas12a, SrMV, RSMV, visual detection, on-site detection

## Abstract

Rapid, sensitive and visual detection of plant viruses is conducive to effective prevention and control of plant viral diseases. Therefore, combined with reverse transcription and recombinase-aided amplification, we developed a CRISPR/Cas12a-based visual nucleic acid detection system targeting sorghum mosaic virus and rice stripe mosaic virus, which cause harm to crop production in field. When the RT-RAA products were recognized by crRNA and formed a complex with LbCas12a, the ssDNA labeled with a quenched green fluorescent molecule will be cleaved by LbCas12a, and then a significant green fluorescence signal will appear. The entire detection process can be completed within 30 min without using any sophisticated equipment and instruments. The detection system could detect samples at a dilution of 10^7^, about 10^4^-fold improvement over RT-PCR, so the system was successfully to detect rice stripe mosaic virus in a single leafhopper, which is the transmission vector of the virus. Finally, the CRISPR/Cas12a-based detection system was utilized to on-site detect the two viruses in the field, and the results were fully consistent with that we obtained by RT-PCR in laboratory, demonstrating that it has the application prospect of detecting important crop viruses in the field.

## 1 Introduction

Viruses are the most prevalent disease-causing agents in plants and pose serious threat to agriculture and food security (Jones and Naidu, 2019). Despite the significant increase of ssDNA viruses in recent years, RNA viruses cause more serious losses in crops and enormously damage to agricultural production (Zhang et al., 2018). The development of a sensitive and accurate diagnostic method is crucial to control plant viruses. A series of methods have been established, including, but not limited to, polymerase chain reaction (PCR), reverse transcription followed by PCR (RT-PCR), loop (or RT-loop) mediated isothermal amplifications (LAMP and RT-LAMP), molecular hybridization, and enzyme-linked immunosorbent assay (ELISA) (Rubio et al., 2020). PCR-based detection methods are the most widely used virus detection technology at present, especially real-time quantitative PCR (qPCR) and (RT-qPCR), which rely on the binding of a virus sequence-specific fluorescent probe to the PCR-amplified region and are become more popular since the epidemic of SARS-CoV-2. However, qPCR and RT-qPCR detection methods require well-equipped laboratories and relatively more expensive reagents, impeding the use of these technologies at resource-limited areas for plant virus diagnosis. ELISA is another widely used technology in virus detection, which has the advantages of high sensitivity and high throughput, but needs viral specific antibodies to the virus structure protein (Clark and Adams, 1977). Most of these methods are faced with some defects such as high cost, limited accuracy, insufficient sensitivity, and inability to use in the field for on-site diagnosis in a low-resource environment. Therefore, establishment of rapid, sensitive and portable sensing of viral detection technologies will greatly promote the research, prevention and control of plant virus diseases.

The clustered regularly short palindromic repeat-associated systems (CRISPR-Cas) comprise sequence-specific RNA-directed endonuclease complexes that bind and cleave nucleic acids (Bhaya et al., 2011). In the last decade, CRISPR-Cas has been extensively exploited in eukaryotic species for genome engineering, molecular immunity, and transcriptional regulation (Doudna and Charpentier, 2014; Zhang et al., 2018; Zhang et al., 2019; Zhao et al., 2020; Zhu et al., 2020). Further, many CRISPR systems were found to unleashes indiscriminate single stranded DNase activity by target identification and cleavage (Li et al., 2018a; Chen et al., 2018). This property can be harnessed for nucleic acid detection, and several detection platforms have been established in the past years. For instance, SHERLOCK (Specific High-sensitivity Enzymatic Reporter UnLOCKing), HOLMES (an one-Hour Low-cost Multipurpose highly Efficient System), and DETECTR (DNA Endonuclease-Targeted CRISPR Trans Reporter) are three pioneer nucleic acid detection platforms, which employ the non-specific endonuclease activities of Cas13-ssRNA reporter or Cas12-ssDNA reporter (Gootenberg et al., 2017; Li et al., 2018b; Chen et al., 2018; Gootenberg et al., 2018; Kellner et al., 2019). These detection platforms have the advantages of high sensitivity and direct display of detection results.

Isothermal amplification techniques, such as loop mediated isothermal amplifications (LAMP) and recombinase polymerase amplification (RPA), are more and more widely used in the detection of viral nucleic acid, because they allow nucleic acid amplification at a single temperature, thus supporting their use in the field for on-site diagnosis in a low-resource environment (Notomi et al., 2000; Piepenburg et al., 2006). LAMP and RPA are further customized as RT-LAMP and RT-RPA when combined with a reverse transcriptase, that can be utilized for the detection of RNA viruses (Fukuta et al., 2003; Euler et al., 2013). In the past few years, LAMP and RPA methods (including RT-LAMP and RT-RPA) have been integrated with CRISPR/Cas system for rapid and portable detection of both DNA and RNA viruses (Kellner et al., 2019; Broughton et al., 2020; Kaminski et al., 2021).

*Sorghum mosaic virus* (SrMV), genus *Potyvirus*, family Potyviridae, is one of the main pathogens causing sugarcane mosaic disease, and is widely distributed in major sugarcane regions in the world at present (Lu et al., 2021). The rapid detection and identification of this virus is helpful to control the sugarcane mosaic disease in the field.

*Rice stripe mosaic cytorhabdovirus* (RSMV) is cytoplasmic rhabdovirus damaging rice production in China (Yang et al., 2017a). RSMV infected plants exhibit distinct symptoms, including yellow stripes, mosaic patterns, and twisted leaf tips (Yang et al., 2017a; Chen et al., 2019), and it is transmitted by *Recilia dorsalis* leafhoppers in a persistent-propagative manner (Yang et al., 2017b). With the increase of the number of leafhoppers in the field in south China, the damage caused by RSMV is also increasing year by year. Therefore, it is necessary to have a method that can detect viruses in the field, and it is better to have the ability to detect viruses in vector insects.

In this study, we developed a rapid, sensitive, specific and visual method to detect SrMV and RSMV based on RT-recombinase-aided amplification (RT-RAA) and CRISPR/Cas12a system. RT-RAA is an isothermal nucleic acid amplification procedure which is similar to RT-RPA. From sample homogenization to RT-RAA followed by CRISPR/Cas12a detection, the entire detection process can be completed within 30 min without using any sophisticated equipment and instruments. The new assay is highly sensitive compared with RT-PCR and highly specific for the target viruses. Furthermore, the method was applied to detect RSMV in samples of a single vector leafhopper, demonstrating that it has the application prospect of detecting important crop viruses in the field.

## 2 Materials and methods

### 2.1 Plant and insect samples

Sugarcane leaves infected with sorghum mosaic virus (SrMV), sugarcane mosaic virus (SCMV), sugarcane yellow leaf virus (SCYLV), and rice plants infected with rice stripe mosaic virus (RSMV), southern rice black-streaked dwarf virus (SRBSDV), rice gall dwarf virus (RGDV), rice ragged stunt virus (RRSV), rice orange leaf phytoplasma (ROLP) were preserved in our laboratory. Leafhoppers (*Recilia dorsalis*) were reared and propagated on the RSMV-infected rice plants. RT-PCR with specific primers of each of these pathogens was conducted to verify the samples were indeed infected.

### 2.2 RT-RAA primer design

The sequences of 11 SrMV isolates were downloaded from NCBI (Supplementary Table S1) and compared by MegAlign (DNAStar, Madison, WI, USA), and the conserved sequences were designed as the RT-RAA primers (Supplementary Table S2). For RSMV, the RT-RAA primers were designed from the conserved region of its encoded N gene (Supplementary Table S2).

### 2.3 RT-RAA assay

The RT-RAA assay was performed using the RT-RAA kit (Cat: S003ZC, ZC Bio-Sci&Tech, Hangzhou, China). The reaction mixture contained 9.5 μl buffer A, 9.5 μl nuclease-free water, 1 μl forward primer (10 μM), 1 μl reverse primer (10 μM), 2 μl buffer B, and 2 μl RNA template. Buffer B was pre-loaded inside the lid, and the reaction tube was centrifuged briefly to ensure the reagents were well-mixed. The products were visualized by gel electrophoresis using 2% agarose gels.

### 2.4 RT-PCR

Total RNA was extracted from leaf samples using the TRIzol reagent (Vazyme, Nanjing, China). RT-PCR amplification was carried out using a One-Step RNA PCR kit (Cat: RR055, Takara, Dalian, China) with the SrMV or RSMV specific RT-RAA primers. The reaction system (10 μl) contains 5 μl of 2X reaction mix, 1 μl RNA template, 0.25 μl forward primer, 0.25 μl reverse primer, and 3.5 μl ddH_2_O. The procedure of PCR was as follows: 50°C for 30 min; 95°C for 5 min; 35 cycles of 95°C for 30 s, 55°C for 30 s, and 72°C for 30 s; and 72°C for 5 min. After amplification, the resulting RT-PCR products were visualized by gel electrophoresis using 2% agarose gels.

### 2.5 The design and synthesis of crRNAs

The crRNAs of LbCas12a recognize a 20-nt target sequence adjacent to a 5′-TTTN-3′ site. The spacer sequence of crRNA was designed to recognize the region between the RT-RAA primers. The spacer sequence was aligned using NCBI BLAST to ensure the specificity of the crRNA target sequence (Supplementary Table S3). The crRNAs were synthesized by Sangon Biotech (Shanghai, China).

### 2.6 Purification of the LbCas12a protein

The LbCas12a was cloned into pET28a (Novagen) with N-terminal 6×His tagging. The plasmid was transformed into *E. coli* strain Rosetta (DE3). For protein expression, a single clone was first cultured overnight in 5-mL liquid LB and then 1% (v/v) inoculated into 200 ml of fresh liquid LB. Cells were grown with shaking at 220 rpm and 37°C until the OD600 reached 0.8, and then 0.5 mM IPTG was added followed by further culture at 37°C for 4 h. Cells were harvested and resuspended in 25 mL lysis buffer (50 mM Tris-Hcl, 600 mM NaCl, 1 mM DTT, and 5% glycerol) with 1 mM PMSF as the protease inhibitor, then were sonicated on ice. The obtained lysate was then centrifuged at 15,000 rpm for 30 min. The supernatant was mixed with 5 mL Ni-NTA beads (ThermoFisher Scientific) and softly shaken for 1 h at 4°C. The beads were washed with wash buffer (lysis buffer supplemented with 30 mM imidazole) and eluted with elution buffer (lysis buffer supplemented with 600 mM imidazole). Fractions containing LbCas12a proteins were verified by SDS-PAGE and then pooled for dialysis with dialysis buffer (50 mM Tris-HCl, 600 mM NaCl, 2 mM DTT) overnight. Finally, the protein was collected and diluted to a final concentration of 10 μM and stored at −80 °C.

### 2.7 The LbCas12a-based fluorescence assay for trans-cleavage activity

The total CRISPR/Cas12a reaction volume was 20 μl, and the reaction mixture contained 500 nM LbCas12a, 500 nM crRNA, 2 μl 10X Enhanced Buffer (100 mM Tris-HCl, 100 mM NaCl, 150 mM MgCl_2_, 10 mM DTT, 5% PEG-200), 2 μl plasmid that contained the target gene, 2 μM fluorophore quencher labeled ssDNA probe (5′-FAM-CCCCCCCC-BHQ1-3′). Reactions were performed at 37°C on multi-functional microplate reader Varioskan LUX (Thermo), and the fluorescence was measured every 30 s (λex: 492 nm; λem: 520 nm). The fluorescence signals were also examined by under a UV Flashlight.

For testing the minimum detection limit of the CRISPR/Cas12a reaction, the RT-RAA products were cloned into pEASY-Blunt Zero vector (TransGen Biotech) and the obtained plasmids were verified by sequencing. Then the concentration of the plasmids was determined by Nanodrop spectrophotometry (Thermo Scientific) and the copy number was calculated. The viral copy number of crude extract was calculated by comparing the Cq number of the plasmids based on RT-qPCR data. The series of 10-fold diluted plasmids or crude extract of virus infected leaves were proceeded for CRISPR/Cas12a reaction system.

### 2.8 Direct virus detection from crude extracts

15–20 mg of leaf tissue or a single insect was homogenized by a hand-held tissue homogenizer in a 1.5-ml tube with 400 μl or 50 μl extraction buffer (6% PEG 200 and 20 mM NaOH), respectively. After incubation at room temperature for 3–5 min, the crude extract was directly subjected to RT-RAA without purification, followed by CRISPR/Cas12a visual detection using the above procedure.

### 2.9 Sensitivity and specificity assay

For sensitivity assay, a crude extract of the leaf sample was considered the 1x dilution. Further 10-fold serial dilutions, 10^1^, 10^2^, 10^3^, 10^4^, 10^5^, 10^6^, 10^7^ and 10^8^ were made and proceed for RT-RAA-CRISPR/Cas12a detection.

For specificity assay, plants infected with different viruses were sampled for RT-RAA-CRISPR/Cas12a detection.

### 2.10 Field sample detection

To test the CRISPR/Cas12a-based detection system, sugarcane and rice samples were collected from different locations in Guangxi, Guangdong. The RT-RAA reaction was incubated at 37°C for 20 min, and 2 µl of the RT-RAA product was used for the CRISPR/Cas12a-based detection as described above. Moreover, to verify the accuracy of the CRISPR/Cas12a-based detection system, the samples were taken back to the laboratory and analyzed by using RT-PCR method.

## 3 Results

### 3.1 Optimization of the conditions for RT-RAA

**FIGURE 1:**
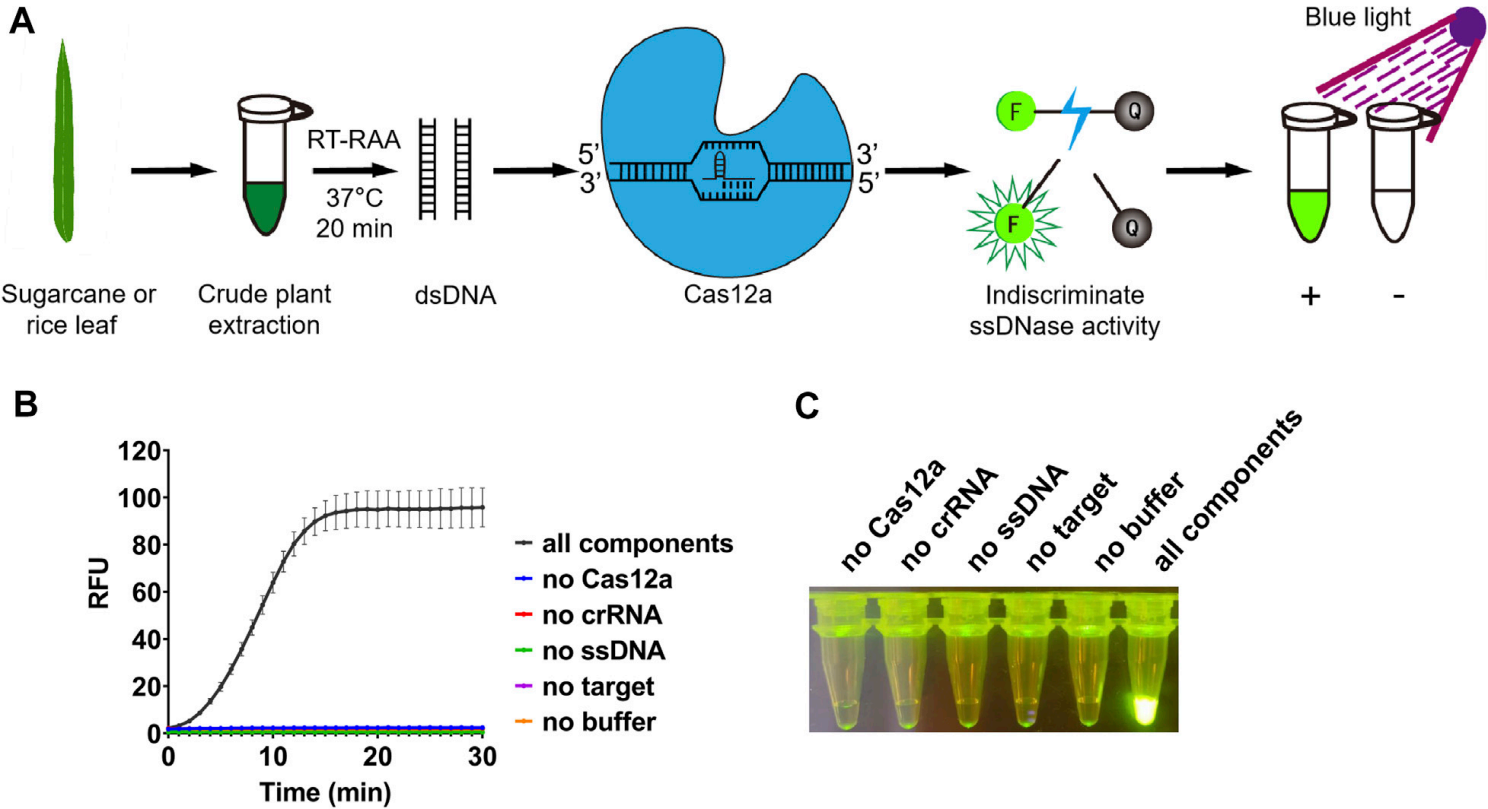
Detection system based on reverse transcription-recombinase-aided amplification and CRISPR/Cas12a. **(A)** Schematic diagram of the method for rapid and visual nucleic acid detection using RT-RAA-CRISPR/Cas12a assay. In this detection system, target gene fragments were amplified with RAA; ssDNA probes (5′-FAM/3′-BHQ1 labeled) were added, and subsequently cleaved by Cas12a to generate green fluorescence. The detection results were directly visible under blue light. **(B)** Validation of the CRISPR/Cas12a assay. In the absence of each component, the real-time fluorescence signal was continuously monitored for 30 min. **(C)** After 30 min of reaction, the tubes were irradiated with UV light for visualization. n = 3 technical replicates, and data points are shown as mean ± SD.

The RT-RAA-CRISPR/Cas12a RNA virus detection platform is shown in Figure 1A. The first step is RT-RAA amplification on the crude extract of the sample. So we optimized the conditions of RT-RAA by using SrMV infected sugarcane leaf samples. We designed multiple RT-RAA primer sets against the conserved region of SrMV genome (Supplementary Table S2). 6 out of 8 sets of primers were effective in the RT-RAA reaction, and the third set (RAA-SrMV-F3 and RAA-SrMV-R3) was more active (Supplementary Figure S1A). We thus proceeded with this primer set in later SrMV RT-RAA experiments. Then we tested the effect of different primers concentration, reaction temperature, and reaction time for the RT-RAA efficiency. The results showed that primer concentration between 0.4–0.8 μM, reaction temperature between 32°C–42°C, and reaction for 20–40 min was suitable for RT-RAA reaction (Supplementary Figure S1B-D). To facilitate and save reagents for the RT-RAA reaction, we set the primer concentration at 0.4 μM, reaction temperature at 37°C, and reaction time for 20 min for further experiments.

### 3.2 Optimization of the conditions for CRISPR/Cas12a-based visual detection

After amplification, the RT-RAA product will be used for detection assay with CRISPR/Cas12a. When the RT-RAA products were recognized by crRNA and formed a complex with LbCas12a, the ssDNA labeled with a quenched green fluorescent molecule will be cleaved by LbCas12a, and then a significant green fluorescence signal will appear (Figure 1A). We purified LbCas12a which was expressed in *Escherichia coli*, and obtained the protein with correct size (Supplementary Figure S2). The detection results can be observed directly under blue light after reacting for approximately 10 min (Figure 1B). In contrast, no fluorescence signal will be produced if the system lacks LbCas12a, crRNA, ssDNA, RT-RAA product, or buffer (Figures 1B, C).

**FIGURE 2:**
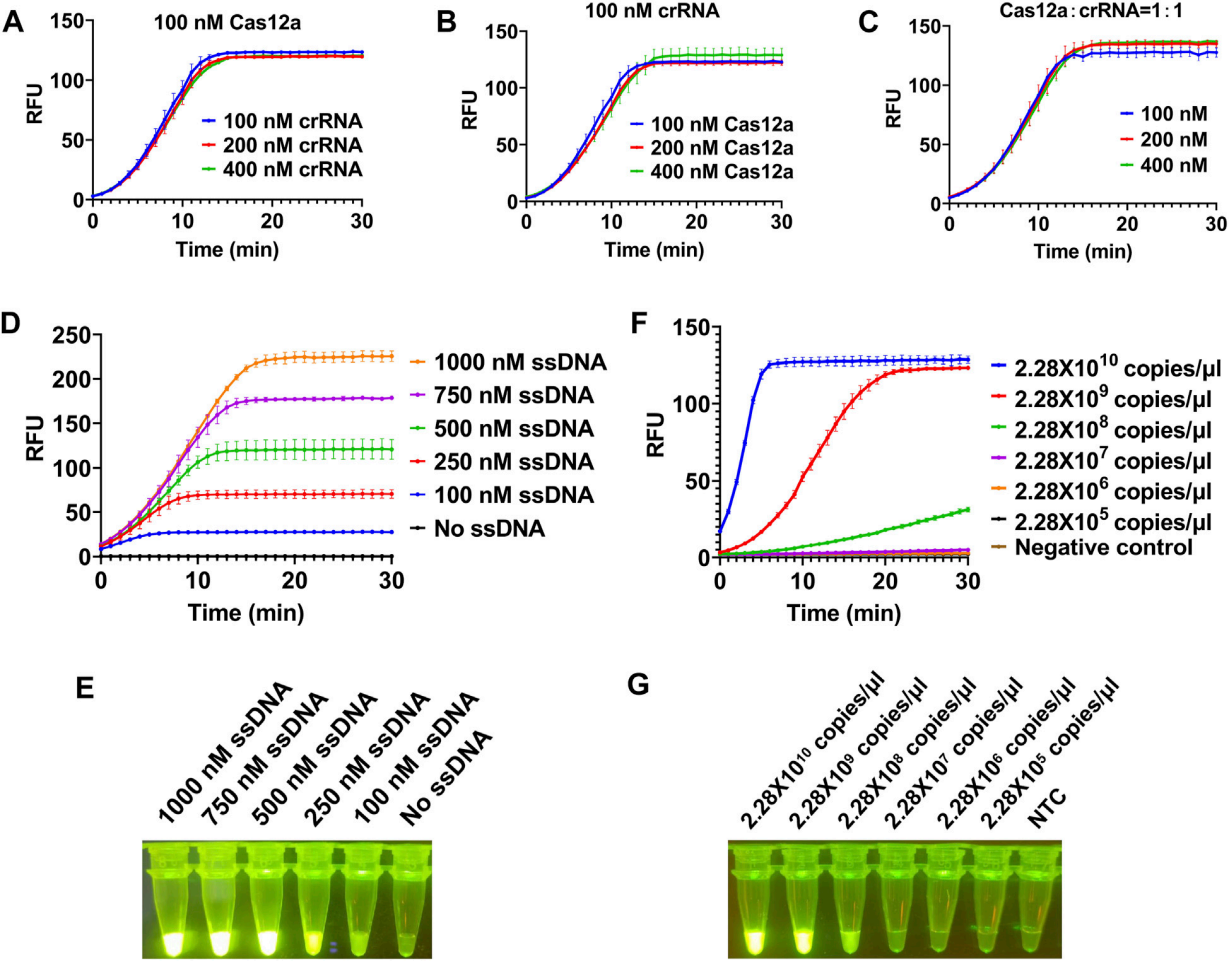
Optimization of reaction conditions for CRISPR/Cas12a-based visual detection. The SrMV RT-RAA product was cloned into vector and the plasmid was used for the optimization of the CRISPR/Cas12a system. **(A)** The concentration of Cas12a protein was constant, and the concentration of crRNA in the system was optimized. **(B)** The concentration of crRNA was constant, and the concentration of Cas12a protein in the system is optimized. **(C)** The concentration of Cas12a protein and crRNA was optimized in equal proportion. **(D)** Real-time fluorescence signal analysis of ssDNA probes at different concentrations. **(E)** Determination of optimal ssDNA concentration by visual observation. **(F)** Real-time fluorescence signal analysis of plasmid templates at different concentrations. **(G)** Determination of optimal plasmid templates concentrations by visual observation. n = 3 technical replicates, and data points are shown as mean ± SD.

To achieve a better detection effect, we optimized the concentration of each component in the system using plasmid as template. We first investigated the dosage of LbCas12a and crRNA in this reaction (100, 200, 400 nM). The results showed that no matter the concentration of LbCas12a or crRNA increased alone or that of both LbCas12a and crRNA was increased, there was no significant improve on the trans cleavage activity of LbCas12a (Figures 2A–C). Next we tested the dosage of ssDNA in this reaction, and a series of concentrations of ssDNA (0, 100, 250, 500, 750, and 1000 nM) were applied. With the increase of the concentration of ssDNA added, the fluorescence signal is gradually enhanced (Figure 2D), and clear green fluorescence was produced when the concentration reached 500 nM (Figure 2E). Together, final concentrations of 100 nM for LbCas12a, 100 nM for crRNA, and 500 nM for ssDNA were used for further experiments.

After determining the optimal concentration of each component in CRISPR/Cas12a system, we subsequently tested the minimum detection limit of this system. We cloned the RT-RAA product into a clone vector and got the concentration of 2.28 × 10^10^ copies/μl plasmid. When the plasmid was diluted to 2.28 × 10^8^ copies/μl, the fluorescence signal could be detected but weak, and when diluted to 2.28 × 10^7^ copies/μl, no obvious fluorescence signal can be detected (Figures 2F,G), indicating the detection limit of our CRISPR/Cas12a system was 2.28 × 10^8^ copies/μl.

### 3.3 Sensitivity and specificity assay for RT-RAA-CRISPR/Cas12a detection for SrMV

**FIGURE 3:**
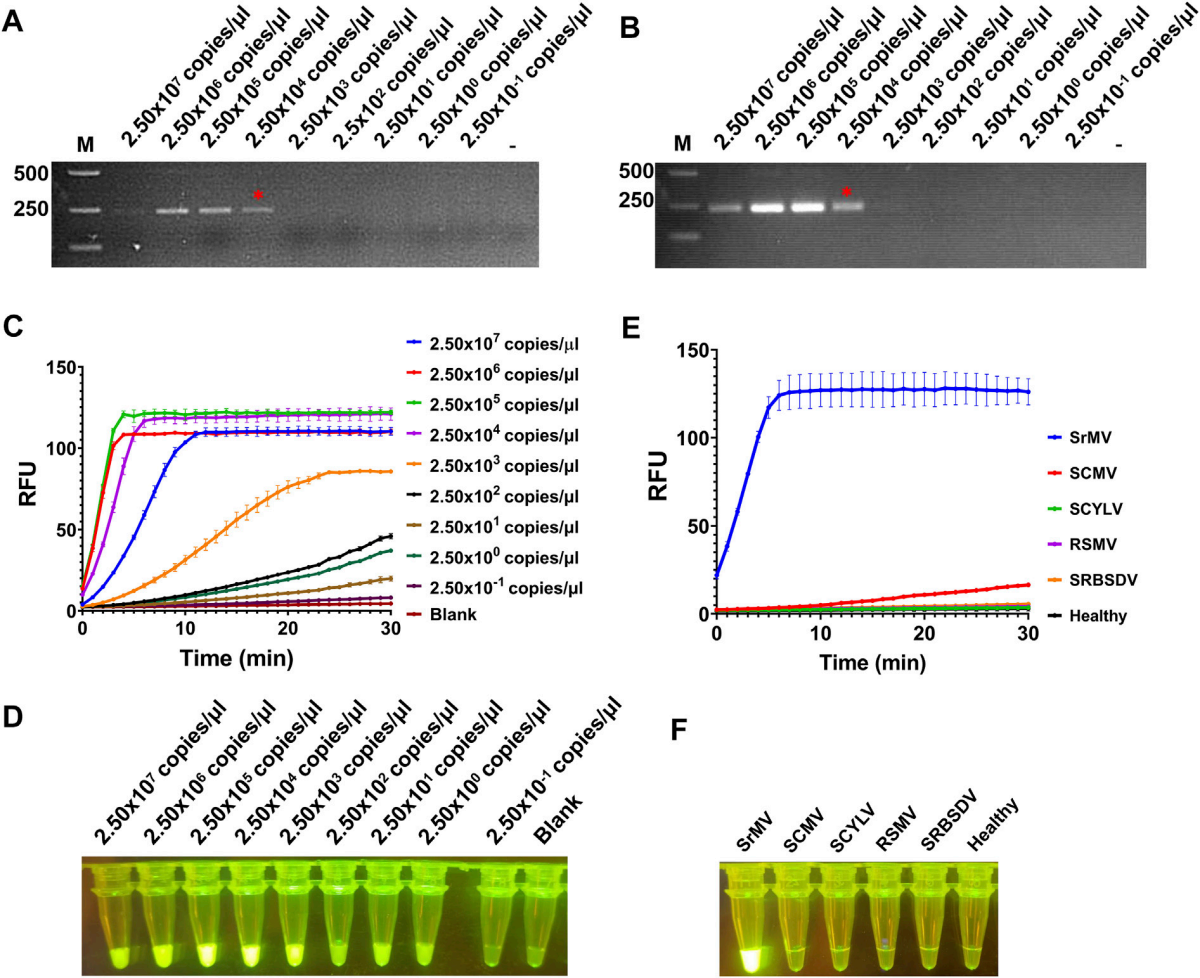
Sensitivity and specificity assay for RT-RAA-CRISPR/Cas12a detection for SrMV. **(A)** The crude extracts of SrMV infected sugarcane leaves were diluted gradiently and tested with RT-RAA. **(B)** The crude extracts were diluted gradiently and tested with RT-PCR. **(C)** The crude extracts were diluted gradiently and tested by RT-RAA-CRISPR/Cas12a detection method, and the real-time fluorescence signal was continuously monitored for 30 min. Water was used as blank control. **(D)** The gradiently diluted crude extracts were tested by RT-RAA-CRISPR/Cas12a method and followed by visual observation. **(E)** The crude extracts of plants samples infected with different viruses were tested by RT-RAA-CRISPR/Cas12a detection method, and the real-time fluorescence signal was continuously monitored for 30 min. Healthy plant sample was used as control. **(F)** The crude extracts of plants samples infected with different viruses were tested by RT-RAA-CRISPR/Cas12a method and followed by visual observation. SrMV: sorghum mosaic virus, SCMV: sugarcane mosaic virus, SCYLV: sugarcane yellow leaf virus, RSMV: rice stripe mosaic virus, SRBSDV: southern rice black-streaked dwarf virus. n = 3 technical replicates, and data points are shown as mean ± SD.

Here we combined our RT-RAA assay and CRISPR/Cas12a detection system together to set up a visual detection for SrMV. To test the sensitivity of the detection method, the crude extract of SrMV infected sugarcane leaves was serially diluted by 10-fold and quantified by RT-qPCR (2.50 × 10^7^ to 2.50 × 10^–1^ copies/μl). The diluted samples were used as templates of RT-RAA or RT-PCR, and then the amplification products were proceeded for electrophoretic detection or CRISPR/Cas12a detection. The results showed that for both RT-RAA and RT-PCR followed by electrophoretic detection, only the samples containing more than 2.50 × 10^4^ copies/μl viral RNA can be detected (Figures 3A, B). In contrast, in the RT-RAA followed by CRISPR/Cas12a detection, fluorescence can still be observed after the crude extract was diluted to 2.50 copies/μl viral RNA (Figures 3C, D). Therefore, the combination of RT-RAA and CRISPR/Cas12a significantly enhanced the sensitivity in virus detection.

Next, we tested the specificity of the RT-RAA-CRISPR/Cas12a method for SrMV detection. We used the crude extracts of plants infected with different viruses as templates to carry out experiments. The results showed that only the samples infected with SrMV could produce strong fluorescence signals, and the others had no obvious fluorescence emitted (Figures 3E, F), indicating the detection method based on RT-RAA and CRISPR/Cas12a was specific.

### 3.4 The development of the CRISPR/Cas12a-based visual detection for RSMV

RSMV is a new emerging rice virus in South China and is lacking rapid on-site detection system (Yang et al., 2017a). We therefore developed the RT-RAA-CRISPR/Cas12a detection system for RSMV. We designed multiple RT-RAA primer sets against the RSMV genome (Supplementary Table S2). 7 out of 8 sets of primers were effective in the RT-RAA reaction, and the first set (RAA-RSMV-F1 and RAA-RSMV-R1) was more active (Supplementary Figure S3A). We thus proceeded with this primer set in later RSMV detection. Then we tested the effect of different primers concentration, reaction temperature, and reaction time for the RT-RAA efficiency. Similar as we did for SrMV detection, primer concentration between 0.1 and 0.4 μM, reaction temperature between 30°C–42°C, and reaction for 20–45 min was suitable for RT-RAA reaction for RSMV (Supplementary Figure S3B-D), and we set the primer concentration at 0.4 μM, reaction temperature at 37°C, and reaction time for 20 min for further experiments.

### 3.5 Sensitivity and specificity assay for RT-RAA-CRISPR/Cas12a detection for RSMV

**FIGURE 4:**
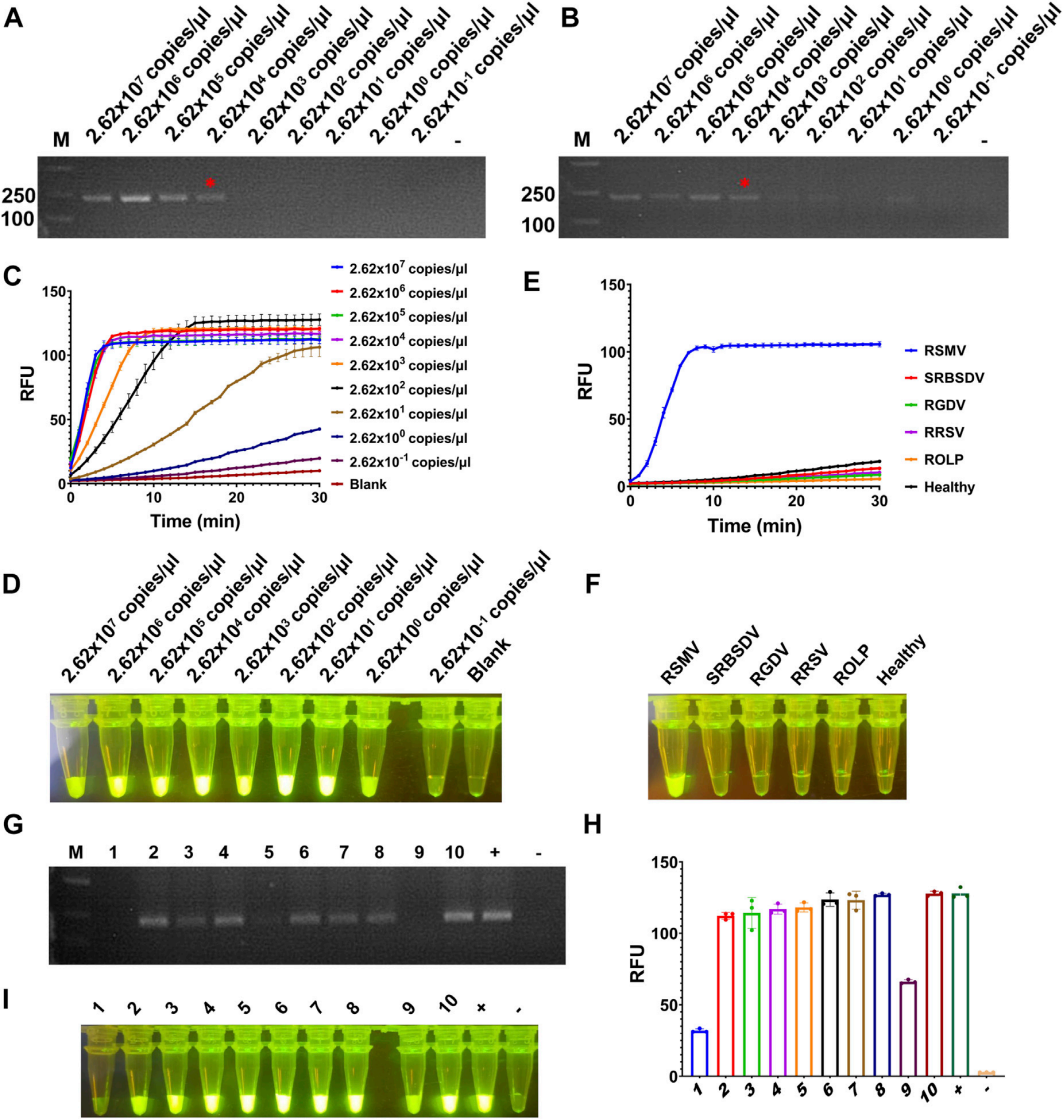
Sensitivity and specificity assay for RT-RAA-CRISPR/Cas12a detection for RSMV. **(A)** The crude extracts of RSMV infected rice leaves were diluted gradiently and tested by RT-RAA. **(B)** The crude extracts were diluted gradiently and tested by RT-PCR. **(C)** The crude extracts were diluted gradiently and tested by RT-RAA-CRISPR/Cas12a detection method, and the real-time fluorescence signal was continuously monitored for 30 min. Water was used as blank control. **(D)** The gradiently diluted crude extracts were tested by RT-RAA-CRISPR/Cas12a method and followed by visual observation. **(E)** The crude extracts of rice samples infected with different pathogens were tested by RT-RAA-CRISPR/Cas12a detection method, and the real-time fluorescence signal was continuously monitored for 30 min. Healthy plant sample was used as control. **(F)** The crude extracts of rice samples infected with different pathogens were tested by RT-RAA-CRISPR/Cas12a method and followed by visual observation. **(G)** The crude extracts of single leafhopper samples were tested by RT-PCR. **(H)** The crude extracts of single leafhopper samples were tested by RT-RAA-CRISPR/Cas12a detection method, and the fluorescence signal was quantitative monitored by microplate reader. **(I)** The crude extracts of single leafhopper samples were tested by RT-RAA-CRISPR/Cas12a detection method and followed by visual observation. RSMV: rice stripe mosaic virus, SRBSDV: southern rice black-streaked dwarf virus, RGDV: rice gall dwarf virus, RRSV: rice ragged stunt virus, ROLP: rice orange leaf phytoplasma. n = 3 technical replicates, and data points are shown as mean ± SD.

To test the sensitivity of the detection for RSMV, the crude extract of RSMV infected rice leaves was serially diluted by 10-fold and quantified by RT-qPCR (2.62 × 10^7^ to 2.62 × 10^–1^ copies/μl). The diluted samples were used as templates of RT-RAA or RT-PCR, and then the amplification products were proceeded for electrophoretic detection or CRISPR/Cas12a detection. The results showed that for both RT-RAA and RT-PCR followed by electrophoretic detection, similar as we did for SrMV, only the samples containing more than 2.62 × 10^4^ copies/μl viral RNA can be detected (Figures 4A, B), but the detection limit of RT-RAA-CRISPR/Cas12a for RSMV go to 2.62 copies/μl viral RNA (Figures 4C, D). We also tested the specificity of the RT-RAA-CRISPR/Cas12a method for RSMV detection. We used the crude extracts of rice plants infected with four different viruses or one phytoplasma as templates to carry out experiments. The results showed that only the samples infected with RSMV could produce strong fluorescence signals, and the others had no obvious fluorescence emitted (Figures 4E, F), indicating this RSMV detection method had high specificity.

RSMV is transmitted by leafhopper vector, and the detection of vector viruliferous rate is the basis for disease occurrence prediction and control (Wang et al., 2021). Hence, we tested the RT-RAA-CRISPR/Cas12a detection for RSMV in leafhopper samples. We picked 10 leafhoppers fed on rice plants infected with RSMV, crude extracted, and detected by RT-PCR or the RT-RAA-CRISPR/Cas12a methods. 7 out of 10 samples showed strong positive signal by both methods, but the last three samples (#1, #5, and #9) only showed obvious signal by RT-RAA-CRISPR/Cas12a system (Figures 4G–I). The results indicate that the RT-RAA-CRISPR/Cas12a visual detection method can effectively detect the leafhopper vector with RSMV, and its sensitivity is higher than that of traditional RT-PCR method.

The application of RT-RAA-CRISPR/Cas12a detection in field.

**FIGURE 5:**
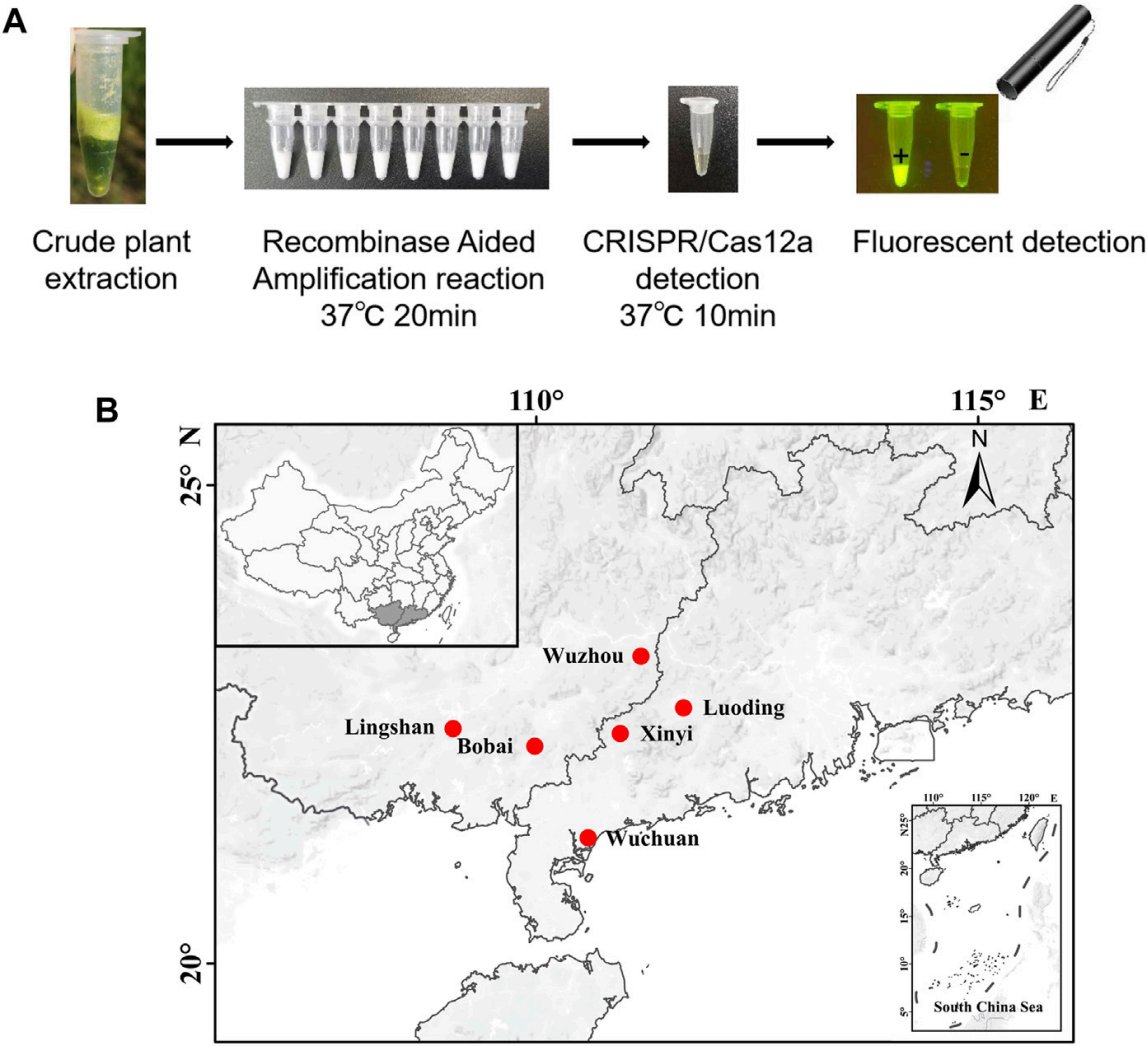
Field on-site detection of SrMV and RSMV by the RT-RAA-CRISPR/Cas12a system. **(A)** The pipeline of on-site plant virus detection based on the RT-RAA-CRISPR/Cas12a system. Take a piece of leaf from the plants and put it into a 1.5 ml tube, followed by grinding with 400 μl crude extraction buffer. Then take 2 μl crude extract into 25 μl RT-RAA reaction system, water bath at 37°C for 20 min. Finally take 2 μl RT-RAA products into 18 μl CRISPR/Cas12a detection solution, water bath at 37°C for 10 min and observed under a UV flashlight. **(B)** The sites we performed the on-site detection of SrMV and RSMV in Guangdong and Guangxi province, China.

According to the above results, we can get a field visual detection process for SrMV and RSMV: 1, Take a piece of leaf from the plants with suspected disease symptoms and put it into 1.5 ml tubes, followed by grinding with 400 μl crude extraction buffer; 2, Take 2 μl crude extract into 25 μl RT-RAA reaction system, water bath at about 37°C for 20 min; 3, Take 2 μl RT-RAA product into 18 μl CRISPR/Cas12a detection system, water bath at about 37°C for 10 min and then observed under a UV flashlight (Figure 5A).

Using this system, we detected over 100 samples for each virus in 6 field locations in South China (Figure 5B). The sugarcane samples were on-site detected for SrMV and the rice samples for RSMV, the infection rates were ranging from 5%–25% of SrMV (Table 1) and 15%–43% of RSMV (Table 2) in different locations. We also take the samples from the same plants back to laboratory for RT-PCR detection, and the detection rate was almost the same (Supplementary Table S4), indicating the RT-RAA-CRISPR/Cas12a on-site visual detection can be effectively applied in the field.

**TABLE 1:** Field samples detection for SrMV by two methods.

Target virus	Location	Samples amount	RT-RAA-CRISPR-Cas12a (on-site)		RT-PCR (in laboratory)	
			No. Positive	Positive rate (%)	No. Positive	Positive rate (%)
SrMV	Luoding	13	3	23.07	2	15.38
	Xinyi	20	1	5.00	1	5.00
	Wuchuan	18	1	5.55	0	0.00
	Xindi	24	5	20.83	5	20.83
	Lingshan	20	2	10.00	1	5.00
	Bobai	16	4	25.00	4	25.00

**TABLE 2:** Field samples detection for RSMV by two methods.

Target virus	Location	Samples amount	RT-RAA-CRISPR-Cas12a (on-site)		RT-PCR (in laboratory)	
			No. Positive	Positive rate (%)	No. Positive	Positive rate (%)
RSMV	Luoding	24	10	41.67	10	41.67
	Xinyi	16	7	43.75	7	43.75
	Wuchuan	12	3	25.00	3	25.00
	Xindi	20	3	15.00	3	15.00
	Lingshan	14	6	42.86	6	42.86
	Bobai	18	4	22.22	4	22.22

## 4 Discussion

Sugarcane diseases caused by SrMV infection are widely distributed worldwide, and rice diseases caused by RSMV infection are becoming more serious and spreading year by year (Yang et al., 2018; Lu et al., 2021). Rapid and accurate detection of these viruses is helpful for forecasting and preventing the epidemics. However, current diagnostics for SrMV and RSMV can be performed only in equipped laboratory, causing a delay between field sample collection and detection results informing. Here, we developed a method for visual detection of RNA virus based on CRISPR/LbCas12a system, including RT-RAA reaction and CRISPR/LbCas12a detection. In this visual detection system, the ssDNA probe, labeled with 5′-FAM and 3′-BHQ1, was added and could be cleaved by the ssDNase activity of LbCas12a to produce green fluorescence. The method is portable and economical, by which the detection results can be observed directly by nacked-eye under blue light. Therefore, this method has a promising application prospect in field on-site detection.

Compared with other plant virus detection methods, this technology shows significant advantages in three aspects. First, the RT-RAA-CRISPR/Cas12a detection system has much higher sensitivity. In our sensitivity assay of RT-RAA-CRISPR/Cas12a system, for both SrMV and RSMV detection had 10^4^ times higher sensitivity than direct RT-RAA or RT-PCR methods. Interestingly, the 10^2^ and 10^3^ diluted crude extract samples showed stronger fluorescence signal than 10° and 10^1^ diluted samples (Figures 3C, D; 4C, D). We speculate that this may be due to the composition of the crude extract buffer affecting the reaction of CRISPR/Cas12a detection system, and suggest diluting the crude extract 100 to 1000 times in use to obtain more sensitive results. Second, the RT-RAA-CRISPR/Cas12a detection system has higher time efficiency. The RT-RAA reaction can be completed within 20 min, and the CRISPR/Cas12a fluorescence display takes only 10 min to observe the results. Therefore, with simple sample collection, homogenization and reagent addition, it only takes about 30 min to get the virus diagnosis results on site. Last but not the least, the RT-RAA-CRISPR/Cas12a detection method has low requirement for equipment and instruments. Unlike traditional PCR assays, RAA reaction does not require repeated heating and cooling process, and the detection results are directly fluorescent displayed in the reaction tube by the CRISPR/LbCas12a system. Thus, this detection system only needs a single temperature water bath (even just a cup of warm water, the RAA reaction does not need accurate temperature) and a UV flashlight.

So far, isothermal amplification combined with CRISPR/Cas12a nucleic acid recognition system has been broadly employed to detect various viruses (Broughton et al., 2020; Duan et al., 2022). Notably, the risk of aerosol contamination during the detection procedure should not be ignored. To avoid transferring the amplified products which easily causes aerosol contamination thus leading to false positive risk, some one-pot methods integrating nucleic acid amplification and CRISPR detection into a single reaction tube have been exploited (Li et al., 2019; Aman et al., 2020; Ding et al., 2020). However, these one-pot methods generally suffer from poor sensitivity due to the cross interference between the reagents of isothermal amplification and CRISPR/Cas12a detection system. Here the detection platform we established was mainly used for on-site virus detection in the field, where has less aerosol contamination than in the laboratory, so a two-step approach was adopted to get best sensitivity of the detection.

In summary, a rapid and sensitive method for visual detection of SrMV and RSMV has been developed. This method for RNA virus detection is highly efficient and its detection limit is much lower than that of RT-PCR. This system has been successfully applied in the field for on-site detection, and the detection rate is almost the same or even higher with this technology compared with RT-PCR detection in the laboratory.

## Data Availability

The raw data supporting the conclusions of this article will be made available by the authors, without undue reservation.
