# Supplementary material for: On-site and visual detection of sorghum mosaic virus and rice stripe mosaic virus based on reverse transcription-recombinase-aided amplification and CRISPR/Cas12a
Source: Front Genome Ed. 2023 Jan 20;5:1124794. doi: 10.3389/fgeed.2023.1124794 (PMC9895793; doi:10.3389/fgeed.2023.1124794)
Supplement: Supplementary file 1 [file DataSheet1.docx]

Supplementary Material

On-site and visual detection of sorghum mosaic virus and rice stripe mosaic virus based on reverse transcription-recombinase-aided amplification and CRISPR/Cas12a

Junkai Wang^1^, Xiuqin Huang^1^, Siping Chen^1^, Jiahao Chen^1^, Zhengyi Liang^1^, Biao Chen^1^, Xin Yang^1^, Guohui Zhou^1^ and Tong Zhang^1,2*^

*** Correspondence:** Tong Zhang: zhangtong@scau.edu.cn

# Supplementary Figures and Tables

For more information on Supplementary Material and for details on the different file types accepted, please see [here](https://www.frontiersin.org/guidelines/author-guidelines#supplementary-material).

## Supplementary Figures


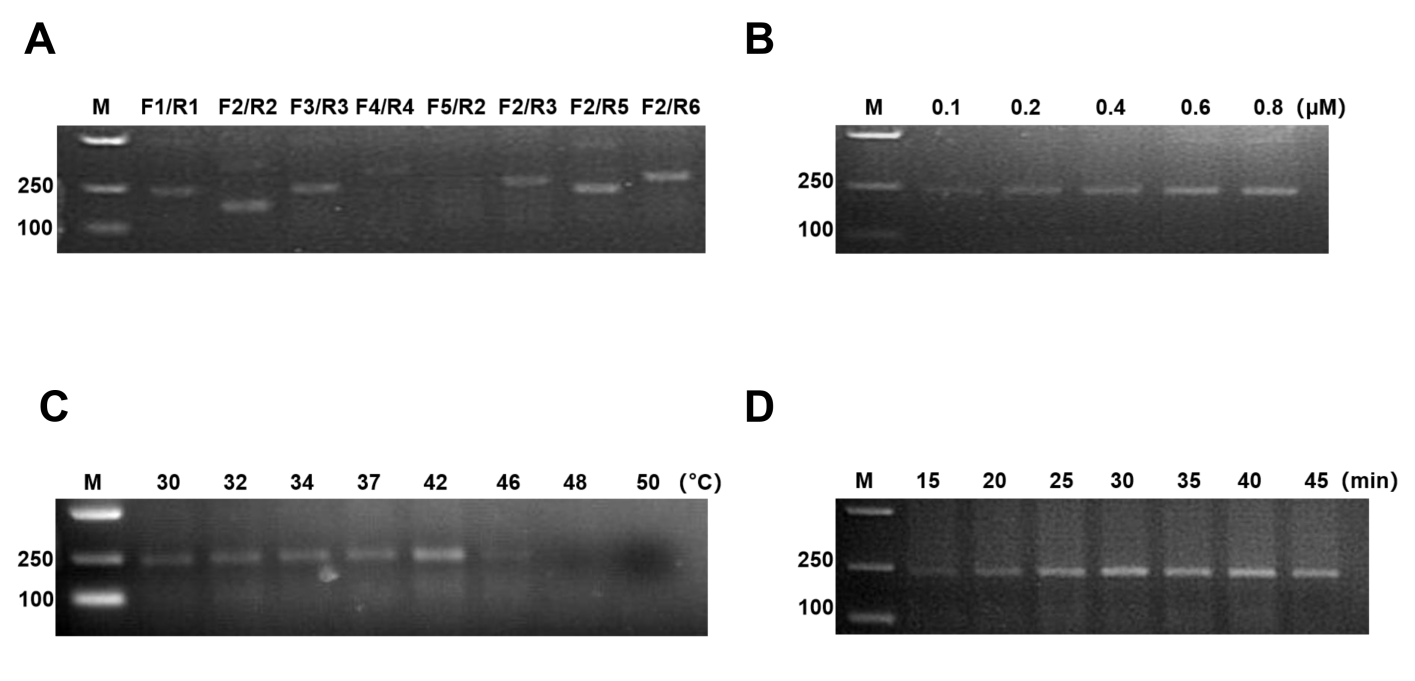


**Supplementary Figure 1.** Establishment of RT-RAA assay for SrMV detection. (A) Different primer pairs were screened for SrMV RT-RAA. The expected size of the RT-RAA products were as follows: F1/R1, 236 bp; F2/R2, 172 bp; F3/R3, 236 bp; F4/R4, 298 bp; F5/R2, 454 bp; F2/R3, 242 bp; F2/R5, 202 bp; F2/R6, 247 bp. (B) Different primer concentrations were tested for SrMV RT-RAA. (C) Different reaction temperatures were tested for SrMV RT-RAA. (D) Different reaction time was tested for SrMV RT-RAA.


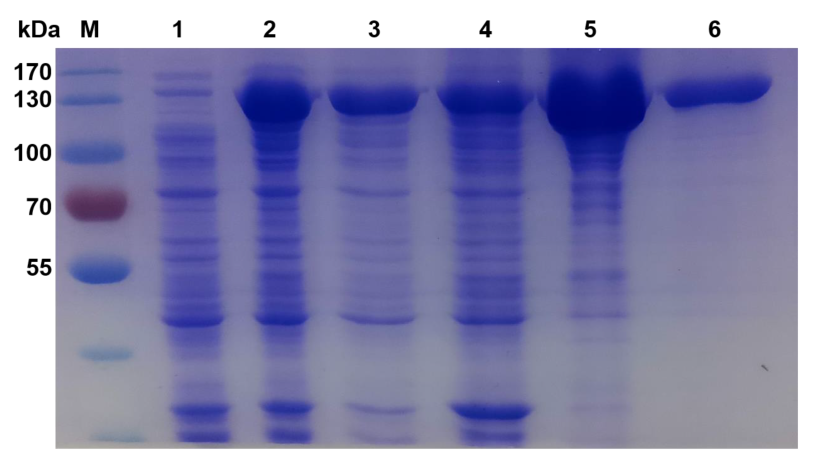


**Supplementary Figure 2.** Purifification of the LbCas12a Protein. SDS-PAGE and coomassie blue staining analysis of purified LbCas12a protein. M: Protein marker. 1: bacterial before adding IPTG. 2: bacterial after adding IPTG for 4 h. 3: Supernatant sample after ultrasonic crushing and high speed centrifugation. 4: The precipitated sample after ultrasonic crushing and high speed centrifugation. 5: Elution of LbCas12a protein after Ni-NTA purification. 6: Elution of LbCas12a protein after ultrafiltration.


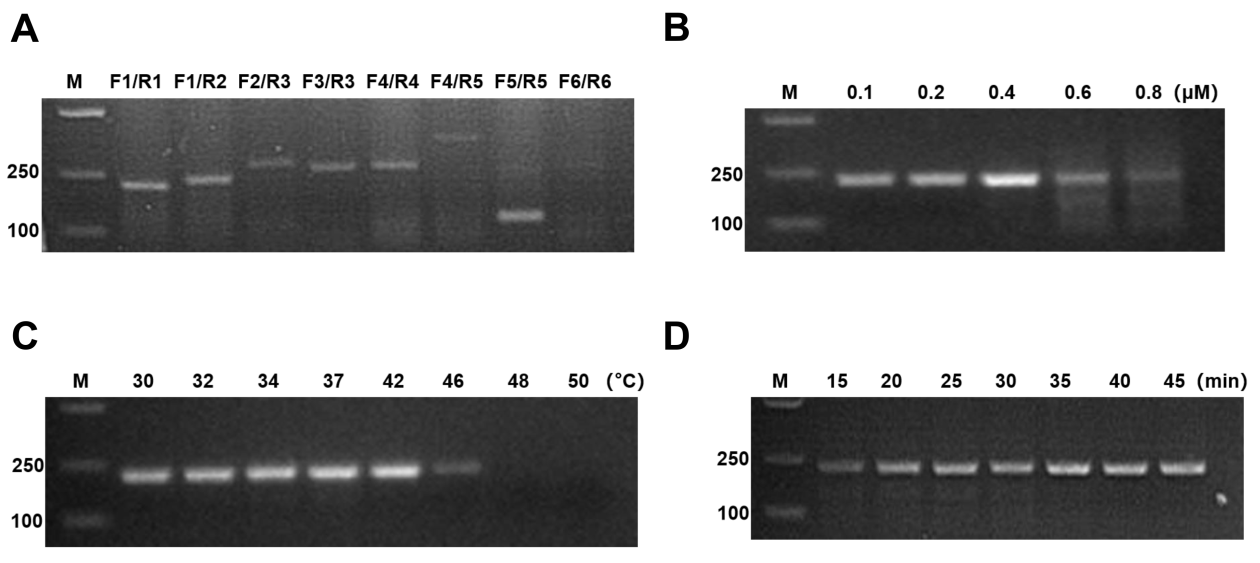


**Supplementary Figure 3.** Establishment of RT-RAA assay for RSMV detection. (A) Different primer pairs were screened for RSMV RT-RAA. The expected size of the RT-RAA products were as follows: F1/R1, 225 bp; F1/R2, 240 bp; F2/R3, 285 bp; F3/R3, 270 bp; F4/R4, 274 bp; F4/R5, 372 bp; F5/R5, 128 bp; F6/R6, 271 bp. (B) Different primer concentrations were tested for RSMV RT-RAA. (C) Different reaction temperatures were tested for RSMV RT-RAA. (D) Different reaction time was tested for RSMV RT-RAA.

## Supplementary Tables

**Supplementary Table 1.** The SrMV genomic sequences used in this study.

| Isolate name | Accession | Sampling location |
| --- | --- | --- |
| FJ-CI20031 | KM025052.1 | Fuzhou, Fujian , China |
| GX-YZ194 | KM025050.1 | Laibin, Guangxi, China |
| GD-YT861 | KM025047.1 | Wengyuan, Guangdong, China |
| GD-ROC222 | KM025046.1 | Suixi, Guangdong, China |
| GX-YT861 | KM025044.1 | Guilin, Guangxi, China |
| GZ-GT11 | KM025042.1 | Wangmo, Guizhou, China |
| GX-LC1362 | KM025041.1 | Laibin, Guangxi, China |
| FJ-GN70 | KM025038.1 | Fuzhou, Fujian, China |
| GX | KJ541740.1 | Guangxi, China |
| FJ-GT53 | KM025043.1 | Fuzhou, Fujian, China |
| GX-YG35 | KM025053.1 | Baise, Guangxi, China |

**Supplementary Table 2.** RAA primer used in this study.

| Oligo names | Sequences (5' to 3') |
| --- | --- |
| RAA-SrMV-F1 | AGTTACGTCGATCTCTTAAACCAAGCATGGGC |
| RAA-SrMV-F2 | ATGGAAAAAAGTTACGTCGATCTCTTAAACCAAGC |
| RAA-SrMV-F3 | AAAAGTTACGTCGATCTCTTAAACCAAGCATGGG |
| RAA-SrMV-F4 | ATGGTGTTTGGACMATGATGGATGGRGAWGA |
| RAA-SrMV-F5 | ATGTCRTAYTGGATAGTKAARAAYCAAGG |
| RAA-SrMV-R1 | CATAAACTGTGGTGGAGTTTGGTTGAAATAGAG |
| RAA-SrMV-R2 | GATGCGTAGCTGAGATATTGTACATTGCGCCT |
| RAA-SrMV-R3 | AAACTGTGGTGGAGTTTGGTTGAAATAGAGCTG |
| RAA-SrMV-R4 | GAACCACGMACTGCTGCDGCYTTCATCTGCAT |
| RAA-SrMV-R5 | TCTCGACTTTTCTGCACTAAARTTYATATTTG |
| RAA-SrMV-R6 | TACATAAACTGTGGTGGAGTTTGGTTGAAATAG |
| RAA-RSMV-F1 | TCTTCTAACTTCTTCTTGACCGATTACCTGCC |
| RAA-RSMV-F2 | ATGAGGCTATCGGAGATGAGGATTCAAAGAAG |
| RAA-RSMV-F3 | ATGAGGATTCAAAGAAGGCTGAGAAGGCTACA |
| RAA-RSMV-F4 | TGCTTACTTGATGAGGCTACTGACCAAAATAG |
| RAA-RSMV-F5 | ATTCTCCGCTATGTGGCTGTCCTCCCTCTT |
| RAA-RSMV-F6 | AACTACTTGATTGGTGCCATGAGGAGCCCTCT |
| RAA-RSMV-R1 | CTTCTTTGAATCCTCATCTCCGATAGCCTCAT |
| RAA-RSMV-R2 | TGTAGCCTTCTCAGCCTTCTTTGAATCCTCAT |
| RAA-RSMV-R3 | CTATTTTGGTCAGTAGCCTCATCAAGTAAGCA |
| RAA-RSMV-R4 | AAGAGGGAGGACAGCCACATAGCGGAGAAT |
| RAA-RSMV-R5 | AGAGGGCTCCTCATGGCACCAATCAAGTAGTT |
| RAA-RSMV-R6 | TAGAGATGTCATCTAATCCAGCAATGTTCATG |

**Supplementary Table 3 .**crRNA used in this study.

| Oligo names | Sequences (5' to 3') |
| --- | --- |
| SrMV-crRNA | UAAUUUCUACUAAGUGUAGAUGGCGCAAUGUACAAUAUCUC |
| RSMV-crRNA | UAAUUUCUACUAAGUGUAGAUCGACGUGACAGAAGUCAAGA |

**Supplementary Table 4.** Detection results of the field samples by two methods.

| Virus for detection | Location | RT-RAA/CRISPR-Cas12a | | | RT-PCR | |
| --- | --- | --- | --- | --- | --- | --- |
|  |  | Positive | Negative | Positive | | Negative |
| SrMV | Luoding | LD1, LD2, LD5 | LD3, LD4, LD6, LD7, LD8, LD9, LD10, LD11, LD12, LD13 | LD1, LD2 | | LD3, LD4, LD5, LD6, LD7, LD8, LD9, LD10, LD11, LD12, LD13 |
|  | Xinyi | XY8 | XY1, XY2, XY3, XY4, XY5, XY6, XY7, XY9, XY10, XY11, XY12, XY13, XY14, XY15, XY16, XY17, XY18, XY19, XY20 | XY8 | | XY1, XY2, XY3, XY4, XY5, XY6, XY7, XY9, XY10, XY11, XY12, XY13, XY14, XY15, XY16, XY17, XY18, XY19, XY20 |
|  | Wuchuan | WC3 | WC1, WC2, WC4, WC5, WC6, WC7, WC8, WC9, WC10, WC11, WC12, WC13, WC14, WC15, WC16, WC17, WC18 | / | | WC1, WC2, WC3, WC4, WC5, WC6, WC7, WC8, WC9, WC10, WC11, WC12, WC13, WC14, WC15, WC16, WC17, WC18 |
|  | Xindi | XD5, XD7, XD18, XD22, XD23 | XD1, XD2, XD3, XD4, XD6, XD8, XD9, XD10, XD11, XD12, XD13, XD14, XD15, XD16, XD17, XD19, XD20, XD21, XD24 | XD5, XD7, XD18, XD22, XD23 | | XD1, XD2, XD3, XD4, XD6, XD8, XD9, XD10, XD11, XD12, XD13, XD14, XD15, XD16, XD17, XD19, XD20, XD21, XD24 |
|  | Lingshan | LS9, LS16 | LS1, LS2, LS3, LS4, LS5, LS6, LS7, LS8, LS10, LS11, LS12, LS13, LS14, LS15, LS17, LS18, LS19, LS20 | LS9 | | LS1, LS2, LS3, LS4, LS5, LS6, LS7, LS8, LS10, LS11, LS12, LS13, LS14, LS15, LS16, LS17, LS18, LS19, LS20 |
|  | Bobai | BB5, BB9, BB12, BB15 | BB1, BB2, BB3, BB4, BB6, BB7, BB8, BB10, BB11, BB13, BB14, BB16 | BB5, BB9, BB12, BB15 | | BB1, BB2, BB3, BB4, BB6, BB7, BB8, BB10, BB11, BB13, BB14, BB16 |
| RSMV | Luoding | LD3, LD4, LD8, LD10, LD14, LD15, LD18, LD19, LD20, LD22 | LD1, LD2, LD5, LD6, LD7, LD9, LD11, LD12, LD13, LD16, LD17, LD21, LD23, LD24 | LD3, LD4, LD8, LD10, LD14, LD15, LD18, LD19, LD20, LD22 | | LD1, LD2, LD5, LD6, LD7, LD9, LD11, LD12, LD13, LD16, LD17, LD21, LD23, LD24 |
|  | Xinyi | XY1, XY5, XY8, XY9, XY11, XY13, XY16 | XY2, XY3, XY4, XY6, XY7, XY10, XY12, XY14, XY15 | XY1, XY5, XY8, XY9, XY11, XY13, XY16 | | XY2, XY3, XY4, XY6, XY7, XY10, XY12, XY14, XY15 |
|  | Wuchuan | WC2, WC5, WC10 | WC1, WC3, WC4, WC6, WC7, WC8, WC9, WC11, WC12 | WC2, WC5, WC10 | | WC1, WC3, WC4, WC6, WC7, WC8, WC9, WC11, WC12 |
|  | Xindi | XD2, XD3, XD17 | XD1, XD4, XD5, XD6, XD7, XD8, XD9, XD10, XD11, XD12, XD13, XD14, XD15, XD16, XD18, XD19, XD20 | XD2, XD3, XD17 | | XD1, XD4, XD5, XD6, XD7, XD8, XD9, XD10, XD11, XD12, XD13, XD14, XD15, XD16, XD18, XD19, XD20 |
|  | Lingshan | LS1, LS3, LS5, LS6, LS8, LS9 | LS2, LS4, LS7, LS10, LS11, LS12, LS13, LS14 | LS1, LS3, LS5, LS6, LS8, LS9 | | LS2, LS4, LS7, LS10, LS11, LS12, LS13, LS14 |
|  | Bobai | BB9, BB12, BB16, BB17 | BB1, BB2, BB3, BB4, BB5, BB6, BB7, BB8, BB10, BB11, BB13, BB14, BB15, BB18 | BB9, BB12, BB16, BB17 | | BB1, BB2, BB3, BB4, BB5, BB6, BB7, BB8, BB10, BB11, BB13, BB14, BB15, BB18 |

The sample name in red indicates the detection result was opposite between the two methods.
